# Buying time: a rationale for examining the use of circadian rhythm and sleep interventions to delay progression of mild cognitive impairment to Alzheimer’s disease

**DOI:** 10.3389/fnagi.2014.00325

**Published:** 2014-12-08

**Authors:** Glenn J. Landry, Teresa Liu-Ambrose

**Affiliations:** ^1^Aging, Mobility, and Cognitive Neuroscience Laboratory, Department of Physical Therapy, Faculty of Medicine, University of British ColumbiaVancouver, BC, Canada; ^2^Djavad Mowafaghian Centre for Brain Health, University of British ColumbiaVancouver, BC, Canada; ^3^Brain Research Centre, University of British ColumbiaVancouver, BC, Canada

**Keywords:** circadian, sleep, chronotherapy, bright light therapy, aging, mild cognitive impairment, Alzheimer’s, dementia

## Abstract

As of 2010, the worldwide economic impact of dementia was estimated at $604 billion USD; and without discovery of a cure or effective interventions to delay disease progression, dementia’s annual global economic impact is expected to surpass $1 trillion USD as early as 2030. Alzheimer’s disease (AD) is the leading cause of dementia accounting for over 75% of all cases. Toxic accumulation of amyloid beta (Aβ), either by overproduction or some clearance failure, is thought to be an underlying mechanism of the neuronal cell death characteristic of AD—though this amyloid hypothesis has been increasingly challenged in recent years. A compelling alternative hypothesis points to chronic neuroinflammation as a common root in late-life degenerative diseases including AD. Apolipoprotein-E (*APOE*) genotype is the strongest genetic risk factor for AD: *APOE*-ε4 is proinflammatory and individuals with this genotype accumulate more Aβ, are at high risk of developing AD, and almost half of all AD patients have at least one ε4 allele. Recent studies suggest a bidirectional relationship exists between sleep and AD pathology. Sleep may play an important role in Aβ clearance, and getting good quality sleep vs. poor quality sleep might reduce the AD risk associated with neuroinflammation and the ε4 allele. Taken together, these findings are particularly important given the sleep disruptions commonly associated with AD and the increased burden disrupted sleep poses for AD caregivers. The current review aims to: (1) identify individuals at high risk for dementia who may benefit most from sleep interventions; (2) explore the role poor sleep quality plays in exacerbating AD type dementia; (3) examine the science of sleep interventions to date; and (4) provide a road map in pursuit of comprehensive sleep interventions, specifically targeted to promote cognitive function and delay progression of dementia.

## Introduction

The economic impact of dementia is staggering. As of 2010 the annual cost of dementia worldwide was estimated at $604 billion USD, equivalent to 1% of global gross domestic product (Wimo and Prince, 2010). Dementia’s economic impact is composed of: (1) direct medical care costs (e.g., clinic visits, drugs, and hospital care); (2) direct social care costs (e.g., dementia support programs and community services); and (3) indirect costs associated with unpaid care (e.g., basic care, and instrumental activities of daily living (ADL; Wimo and Prince, 2010). Indirect costs—$252 billion USD, contributing 42% of dementia’s economic impact—are a heavy burden borne almost entirely by friends and family of people living with dementia (Wimo and Prince, 2010). Dementia’s drag on the global economy increases dramatically as a function of disease severity: (1) the economic impact doubles as dementia progresses from mild to severe; and (2) there is a four-fold increase in cost for institutionalization compared to community dwelling (Prince and Jackson, 2009). However, an aging population is the greatest driver increasing dementia’s prevalence and economic impact. Current projections estimate the number of people living with dementia will reach 65.7 million by 2030 and 115.4 million by 2050 (Prince et al., 2013a). Without discovery of a cure or development of effective interventions to prevent or delay disease progression, dementia’s annual worldwide economic impact is expected to surpass $1 trillion USD as early as 2030 (Wimo and Prince, 2010; Prince et al., 2013b).

Decades of dementia research have made two things abundantly clear: (1) a cure is likely many years away; and (2) the inescapable economic reality is that society desperately needs interventions specifically targeting individuals at high risk for dementia. Effective interventions could bridge the gap, decreasing dementia’s economic burden, while buying time and conserving the resources necessary to develop a cure. To date, lifestyle interventions to promote cognitive function in older adults at risk for dementia have often focused on exercise (Liu-Ambrose et al., 2010, 2012; Anderson-Hanley et al., 2012; Barnes et al., 2013; Nagamatsu et al., 2013) or cognitive training (Basak et al., 2008). However, recent findings (Lim et al., 2013) suggest sleep quality plays a critical role in preserving cognitive function and reducing the risk of dementia in individuals with a genetic susceptibility to Alzheimer’s disease (AD)—the most common cause of dementia. The importance of sleep for human health and performance has long been well established empirically (Van Dongen et al., 2003; Walker, 2008; McCoy and Strecker, 2011; Caruso, 2014), and yet sleep is typically the first sacrifice made in order to meet the demands of daily schedules in a 24-h society, as articulated nicely in the book “Sleep: a very short introduction” (Lockley and Foster, 2012). The growing popularity of “energy drinks” may be reflective of a disturbing societal trend toward increased sleep debt and heightened daytime sleepiness. The current review aims to: (1) identify individuals who are at high risk for dementia and who may benefit most from sleep interventions; (2) explore the role poor sleep quality plays in exacerbating AD type dementia; (3) examine the science of sleep interventions to date; and (4) provide a road map in pursuit of comprehensive sleep interventions, specifically targeted to promote cognitive function and delay progression of dementia.

## Dementia: Alzheimer’s disease and *APOE*-genotype

Dementia, literally meaning devoid of the mind, is a clinical syndrome characterized by a persistent and typically progressive state of degraded cognitive function (e.g., learning, memory, executive function and decision making) serious enough to impair occupational or social functioning (Apostolova et al., 2012). Dementia has numerous causes commonly classified as either neurodegenerative (e.g., Alzheimer’s, Parkinsonian, or Frontotemporal dementias) or non-degenerative (e.g., Vascular, Multiple Sclerosis, or Alcoholic dementias) (Apostolova et al., 2012). Alzheimer’s disease is by far the most common cause of dementia, accounting for as many as 75% of all cases (Prince and Jackson, 2009). It is the 5th leading cause of death in the United States for adults 65 years or older (Thies and Bleiler, 2013). Alzheimer’s disease pathogenesis is thought to result from synaptic failure as a function of interference in synaptic transmission and ultimately synaptic loss (Marcello et al., 2008; Nimmrich and Ebert, 2009; Selkoe, 2011). Historically, AD pathology has been characterized by neuronal cell death and brain atrophy—hallmarked by the accumulation and deposition of amyloid plaques and neurofibrillary tangles (NFTs)—in limbic and association cortices as well as related subcortical nuclei (Selkoe, 2011). More recently, histopathological analysis of over 2300 postmortem brains ranging in age from 1–100 years suggests AD has a much more nuanced and staged pathological process than previously expected, with an extended preclinical period and disease course that may span five or more decades (Braak et al., 2011). If so, the window for targeted interventions to combat AD progression may be much larger than previously imagined. Clearly intervention early in the pre-clinical stages would be best.

### The amyloid hypothesis of Alzheimer’s disease

The discovery in 1984 of the amino acid sequence for amyloid-beta (Aβ; Glenner and Wong, 1984b) and subsequent linking of Aβ to AD (Glenner and Wong, 1984a) gave rise to the “amyloid hypothesis”; which contends that AD results from toxic accumulation of Aβ, either by overproduction or some clearance failure, altering membrane protein function and interfering with synaptic transmission, ultimately causing neuronal cell death (Tanzi and Bertram, 2005; Selkoe, 2011, 2013). The amyloid hypothesis gained credence with the discovery that the apolipoprotein E (*APOE*) genotype is the strongest genetic risk factor for AD (for reviews see Kim et al., 2009; Holtzman et al., 2012). Apolipoprotein E is a polymorphic gene—with three common alleles (ε2, ε3, and ε4)—that plays a central role in lipid metabolism and transport as well as cholesterol absorption from the intestine (for reviews see Seripa et al., 2011; Egert et al., 2012). Individuals with the *APOE*-ε4 allele are predisposed to accumulate Aβ (Castellano et al., 2011) and are at significantly higher AD risk (Poirier et al., 1993; Strittmatter et al., 1993), whereas the ε2 allele is protective against AD (Corder et al., 1994). Recently, a meta-analysis pooled data collected from over 27,000 patients in 33 countries, and examined prevalence of the ε4 allele among patients diagnosed with AD (Ward et al., 2012b). Their findings indicate that 48.7% of AD patients have at least one ε4 allele, while 9.6% are homozygous-ε4/ε4. In addition to increasing AD risk, the *APOE*-ε4 genotype likely plays a role in accelerating AD onset (Roses, 1996). Age has long been considered the greatest AD risk factor: after the age of 65, prevalence and incidence of AD doubles with each 5-year increment in age (Jorm and Jolley, 1998; Brookmeyer et al., 2007; Prince et al., 2013a). Interestingly, the ε4 population distribution provides a genetic basis for variance in age of AD onset, as evidenced by an analysis of AD age of onset as a function of *APOE* genotype: the mean age of onset varies from less than 70 years for ε4/ε4 to over 90 years for ε3/ε2 (Roses, 1996). Taken together, these findings establish the importance of *APOE* genotype in AD and appear to provide a compelling case for the amyloid hypothesis.

The ε4 allele is associated not only with increased AD risk, but also increased plasma levels of cholesterol (Ehnholm et al., 1986; Boerwinkle et al., 1987; Fenili and McLaurin, 2005), as well as higher rates of cardiovascular disease (Song et al., 2004; Anoop et al., 2010) and stroke (Kalaria, 2001; Szolnoki and Melegh, 2006). Furthermore, the ε4 allele amplifies AD risk associated with diabetes. For example type 2 diabetes mellitus (T2DM) is a known independent risk factor that almost doubles a person’s AD risk (Peila et al., 2002), but that risk escalates even further in individuals with T2DM and the ε4 allele; specifically, individuals with both T2DM and the ε4 allele are more than 5 times more likely to develop AD than people with neither risk factor (Peila et al., 2002). In addition, the ε4 allele alters brain response to acute injury following insults such as hemorrhagic stroke, exacerbating neuronal injury and hindering repair processes (Tang et al., 2003; Lanterna and Biroli, 2009). Given the risks associated with the ε4 allele, one might expect this allele to have been selected out or at least be rare and yet prevalence of the *APOE*-ε4 genotype for most of the world’s ethnicities falls between 10–20%, with a range of 6–40% (Corbo and Scacchi, 1999; Singh et al., 2006).

By itself the ε4 allele does not doom one to certain death by AD or vascular disease, nor does the accumulation of plaques and tangles lead to dementia in all cases. Post mortem analyses of brains have uncovered many cases in which AD type plaques and tangles are present without the expected deficits in cognitive function (Davis et al., 1999; Price et al., 2009; Balasubramanian et al., 2012). Conversely, in studies of very old adults—individuals 80 to 100+ years old—up to 50% of dementias previously diagnosed as AD were later determined to be of unknown etiology (i.e., postmortem analysis failed to identify brain pathology typical of AD or other dementias; Crystal et al., 2000; Imhof et al., 2007; Middleton et al., 2011). So now, almost three decades after the birth of the amyloid hypothesis, it would seem that deposition of Aβ plaques is neither necessary nor sufficient to produce dementia characteristic of AD. Indeed it now appears that when compared with Aβ pathology, severity of NFT pathology—specifically in the neocortex—is more predictive of the extent of cognitive impairment. Neurofibrillary tangles have traditionally been considered downstream from Aβ plaque deposition; however, recent evidence suggests NFT precursors take place long before Aβ plaque deposition (Braak et al., 2011). In light of these findings, the amyloid hypothesis has been increasingly challenged in recent years (Reitz, 2012; Balin and Hudson, 2014; Drachman, 2014). For example, a compelling alternative hypothesis suggests late-onset AD is the result of chronic inflammatory conditions linking AD with vascular disease of small blood vessels in the brain—induced by oxidative inflammation and dysregulated amyloid metabolism (Marchesi, 2011; Krstic and Knuesel, 2013). A neuroinflammatory perspective of AD is discussed in the next section.

Critics of the amyloid hypothesis see Aβ plaques and NFTs as markers of AD, downstream in the disease process but not the root cause; whereas proponents of the amyloid hypothesis point to theoretical constructs such as “cognitive reserve” (i.e., a conceptual framework accounting for individual differences in capacity to withstand neural insults such as brain injury or pathology (Stern, 2009)) and other genetic, lifestyle, or environmental factors that may serve to moderate one’s AD risk. These moderators are thought to include: protective genotypes (e.g., *APOE*-ε2 (Corder et al., 1994; Tanzi and Bertram, 2005)), good sleep quality (Lim et al., 2013), cardiovascular health, education, and other hallmarks of a healthy lifestyle such as life-long learning, exercise and eating well (Imtiaz et al., 2014). The underlying mechanisms by which these moderators preserve cognitive function—despite accumulation of plaques and tangles normally associated with AD—have yet to be fully explained. It is clear, however, that individuals with the *APOE*-ε4 genotype are at high risk for AD type dementia, and may represent an ideal target for interventions.

### The neuroinflammatory perspective of Alzheimer’s disease

An alternative to the classic amyloid centric view of AD suggests that late-onset AD results from age-related alterations in innate immunity and chronic systemic inflammation (for review see Krstic and Knuesel, 2013). According to this perspective, a persistent inflammatory state leads to dysregulation of clearance mechanisms of misfolded or damaged neuronal proteins, and to tau-associated impairments of axonal integrity and transport (Krstic and Knuesel, 2013). In line with this perspective, growing evidence points to Aβ plaques as a downstream consequence of prior changes—possibly related to chronic systemic inflammation—leading to neuronal and synaptic losses (for review see Drachman, 2014). Importantly, accumulation of Aβ plaques and NFTs are widely recognized as proinflammatory processes (Finch, 2011). Thus, it is possible that these hallmarks of AD pathology originate from chronic systemic inflammation—as a function of aging combined with proinflammatory genetic and lifestyle factors (for review see Watt, 2014)—but then serve to exacerbate neuroinflammation and thereby accelerate AD onset and progression. As such, accumulation and/or the clearance failure of Aβ plaques and NFTs may be more related to chronic inflammation than previously recognized (Krstic and Knuesel, 2013; François et al., 2014).

Indeed, evidence is growing in support of a neuroinflammatory perspective of AD—which acknowledges amyloid as a critical component in the disease process—but sees much of the damage in AD to be better associated with the effects of chronic inflammation (for review see Akiyama et al., 2000). Interestingly, most of the polymorphisms conferring increased late-onset AD risk are located in genes of the innate immune system (Harold et al., 2009; Lambert et al., 2009). An intriguing alternative explanation for increased AD risk associated with the *APOE*-ε4 allele is that it is the most proinflammatory *APOE* allele. Furthermore, it appears that chronic systemic inflammation may provide a common underlying link connecting many AD risk factors (i.e., *APOE* genotype, cardiovascular disease, T2DM, poor diet, poor sleep quality, and a sedentary lifestyle)—all of which may be considered proinflammatory factors (Watt, 2014).

## Sleep in normal aging and Alzheimer’s disease

Sleep changes as a function of normal aging both in terms of decreased quality and quantity (for reviews see Espiritu, 2008; Crowley, 2011). Sleep complaints are common in older adults: more than 55% of adults 65 years or older have at least one chronic sleep complaint (Foley et al., 1995). The most common being an inability to stay asleep at night, followed closely by chronic complaints of excessive daytime sleepiness, resulting in frequent daytime naps (Foley et al., 1995). These complaints, in particular the reports of excessive daytime sleepiness (a key indicator of accumulated sleep debt (Carskadon et al., 1986; Johns, 1991)), suggest that age-related changes in sleep are likely the result of something beyond reduced need for sleep. If age-related changes in sleep were simply the result of reduced need, then why would the majority of older adults complain about decreased quality and quantity of their sleep? Whether these changes in sleep should even be considered “normal” is well worthy of debate, but for the purposes of this review, suffice it to say sleep is often disrupted as a function of seemingly normal aging.

By comparison, sleep disruptions in AD are exaggerated to the point that they could be characterized as a form of accelerated or hyper-aging: sleep disruptions in adults with AD are similar in nature to those observed in age-matched adults without AD, but tend to be more extreme and progress faster (for review see Bliwise, 1993). Nighttime sleep in AD is repeatedly interrupted by bouts of restlessness and active periods of wakefulness; whereas daytime activity is routinely broken up by sleep intrusions (Satlin et al., 1995). As AD progresses, day and night become increasingly difficult to distinguish in terms of behavioral sleep-wake rhythms (as measured by actigraphy; van Someren et al., 1996; Hatfield et al., 2004; Fetveit and Bjorvatn, 2006). For older adults with moderate to severe AD, extreme disruption of the sleep-wake rhythm is typical; to the extent that their disrupted sleep significantly increases the burden on family members and caregivers (McCurry et al., 1999)—an important topic that will be discussed at length in a later section.

### Sleep mechanisms

To better understand the nature of sleep disruptions in aging and AD, a brief review of the biology that drives sleep-wake rhythms is instructive (Dijk and Czeisler, 1995; Mistlberger, 2005; Beersma and Gordijn, 2007; Chokroverty and Avidan, 2012). There are two distinct mechanisms that determine sleep need, the timing of sleep onset, and sleep duration (Daan et al., 1984; Borbély et al., 1989): (1) a homeostatic recovery process that increases sleep need as a simple function of prior wakefulness; and (2) a circadian mechanism (i.e., ~24 h biological clock, see Moore, 2013) that coordinates physiology and behavior with the solar day-night cycle (for review see Golombek and Rosenstein, 2010). The homeostatic recovery process is gated by the circadian mechanism (Daan et al., 1984), which thereby extends and consolidates physiological states (i.e., sleep or wakefulness). Beyond its role in gating the homeostatic sleep drive, the circadian mechanism actively drives both states—sleep and wake—in order to coordinate physiology and behavior with the solar cycle (for review see Mistlberger, 2005). This process of synchronizing internal physiology and behavior with the solar cycle is called entrainment.

In humans, the net result of these combined mechanisms is a biphasic diurnal sleep-wake rhythm synchronized with the 24 h solar cycle—meaning the ~24 h rhythm is normally composed of a single wake phase of ~16 h, with the majority of activity occurring during the day; and a single sleep phase of ~8 h at night (Beersma and Gordijn, 2007). The circadian “clock” consolidates sleep and wake states in part by driving two “zones” or gates of behavioral state that oppose the homeostatic mechanism (Lavie, 1986; Dijk and Czeisler, 1995): (1) a wake maintenance zone that occurs 2–3 h prior to habitual sleep onset—a time when homeostasis drives hard for sleep due to the extended duration of prior wakefulness; and (2) a sleep maintenance zone that occurs roughly 2–3 h prior to habitual wake onset—when several hours of prior sleep have drained the homeostatic sleep drive (Strogatz et al., 1986; Dijk and Czeisler, 1995). By delaying sleep onset in the early evening, the wake maintenance zone intensifies the homeostatic sleep drive such that when this zone ends and the circadian sleep gate opens, the transition from wake to sleep is very rapid (typically within 10–20 min; Carskadon et al., 1986). Conversely, in the early morning—after several hours of sleep have drained the homeostatic sleep drive—the circadian clock boosts sleep drive to extend the sleep phase and maximize sleep efficiency (i.e., the time spent actually asleep vs. time spent in bed between sleep and wake states). This sleep maintenance zone occurs at the nadir of the daily body temperature rhythm (T_b_-min: the lowest point in the core body temperature rhythm, which typically occurs ~2–3 h prior to habitual activity onset) and the peak of rapid-eye movement (REM) sleep (Dijk and Czeisler, 1995), which is important for consolidation of learning and memory (Walker and Stickgold, 2004; Stickgold, 2005).

Circadian gating of the homeostatic recovery sleep process is an elegant solution to the fragmentation of sleep-wake rhythms that would otherwise occur if homeostasis alone were to determine sleep drive. As such, age-related circadian dysregulation and impaired circadian gating of the homeostatic sleep drive could explain disrupted sleep-wake rhythms typically observed in older adults (Münch et al., 2005; Cajochen et al., 2006).

### Circadian rhythms and sleep in normal aging

A detailed survey of circadian rhythms and aging is beyond the scope of the current review; however age-related changes of the circadian system have been well characterized elsewhere (for reviews see Monk and Kupfer, 2000; Touitou and Haus, 2000; Weinert, 2000; Hofman and Swaab, 2006; Farajnia et al., 2014). In short summary, the suprachiasmatic nucleus (SCN; a bilateral set of hypothalamic neurons situated above the optic chiasm) serves as the biological clock; and much like other areas of the brain, the SCN is vulnerable to the aging process (Touitou and Haus, 2000; Hofman and Swaab, 2006). Age-dependent changes in the human SCN include decreased volume and cell number (Swaab et al., 1985; Hofman, 1997), decreased amplitude and disrupted output signaling (i.e., peptide expression (Hofman and Swaab, 1994)), and decreased sensitivity to input signals such as light (i.e., for moderate light intensity (Duffy et al., 2007) but not bright light (Benloucif et al., 2006)).

Age-related change in light sensitivity is particularly important since light is the primary stimulus the SCN uses to coordinate physiology and behavior with the solar cycle (Monk and Kupfer, 2000; Weinert, 2000). As such, age-dependent changes of the circadian system and weakened circadian signaling likely play a prominent role in the fragmentation of sleep-wake rhythms observed in older adults. For example, early awakenings and the inability to sustain sleep through the early morning hours may be the result of impaired functioning of the circadian sleep maintenance zone. Timing of the sleep maintenance zone is synchronized with T_b_-min (~4:00 am for individuals who usually wake-up ~7:00 am). In young-middle aged adults this zone is associated with extreme propensity for sleep and it is typically difficult to wake people at this point of their sleep phase (Lavie, 1986). Furthermore, even when they are awakened, most people find it very easy to fall back to sleep during the sleep maintenance zone. By contrast, arousal thresholds in older adults are much lower around T_b_-min and once awakened, older adults may have more difficulty falling back to sleep after their T_b_-min (Phillips and Ancoli-Israel, 2001). However, the latter point is not without controversy given findings that suggest older adults wake up more often than younger adults, but are able to fall asleep at the same rate (Klerman et al., 2004).

Another age-related sleep characteristic is the tendency for older adults to go to bed earlier than they did when they were younger (based on self-report measures (Roenneberg et al., 2007)). On average, older adults go to bed ~2 h earlier than younger adults relative to clock time (Roenneberg et al., 2007)—which could correspond with the circadian wake maintenance zone (Lavie, 1986); however assessments comparing circadian phase of sleep-onset are necessary to confirm this possibility. Nonetheless, quantitative assessment of wake maintenance zone strength has confirmed age-dependent weakening (Münch et al., 2005): compared with young adults (20–31 years), older adults (57–74 years) sleep significantly more during the wake maintenance zone and report higher subjective sleepiness ratings in the late afternoon and evening (Münch et al., 2005). Excessive daytime sleepiness—the second most common chronic sleep complaint in older adults—could also be explained by impaired functioning of the circadian daytime arousal/wakefulness signal (Mistlberger, 2005). As such, it seems likely that typical sleep disruptions in older adults are due to age-related changes in the circadian system, resulting in greater homeostatic influence on sleep drive. Therefore, interventions targeting enhanced functioning of circadian gated wake-maintenance and sleep-maintenance zones may be an effective strategy to improve sleep quality in older adults.

### Circadian rhythms and sleep in Alzheimer’s disease

Sleep disruptions in AD are exaggerated in a way that implies a form of accelerated or hyper-aging (Witting et al., 1990). The fragmentation of sleep-wake rhythms in AD patients is more similar to much older adults without AD than age-matched adults without AD. This notion of hyper-aging in AD is supported by analysis of SCN atrophy in AD vs. non-AD (Swaab et al., 1985; Zhou et al., 1995; Stopa et al., 1999). Antibody-staining of arginine-vasopressin (AVP) expressing SCN neurons shows age-dependent decreases in volume and cell number; however SCN atrophy doubles in age-matched adults with AD vs. non-AD (Swaab et al., 1985). Importantly, in discussing this study’s findings, the authors point out that the 50% reduction in AVP-expressing SCN cells—observed in adults with AD—matches the lesion magnitude required to disrupt circadian rhythms in rodent studies (Van den Pol and Powley, 1979; Pickard and Turek, 1983). Arginine-vasopressin (AVP) mRNA expression in the human SCN provides additional evidence that sleep disruptions in AD are likely caused in part by changes within the SCN (Liu et al., 2000). This study showed AVP mRNA expression is three times lower in AD patients than controls matched for age and time of death. So to summarize the science to date, it is clear that sleep is disrupted in normal aging, these disruptions are exaggerated in AD, and that circadian dysregulation is likely an underlying mechanism of disrupted sleep-wake rhythms in both normal aging and AD. These findings raise an important question: are age-related changes in sleep-wake rhythms clinically relevant to AD, beyond the inconvenience, discomfort, or frustration these disruptions may cause patients and caregivers?

## Getting quality sleep is critical for older adults at risk for Alzheimer’s disease

**Figure 1:**
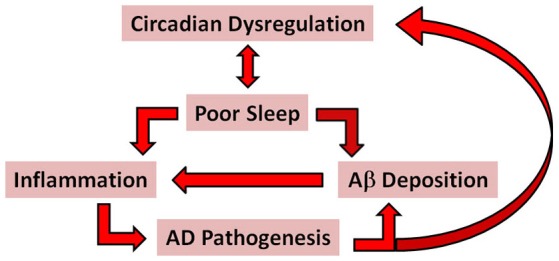
**A simplified schematic of the proposed pathways associated with AD pathogenesis**. The red arrows indicate possible pathways whereby circadian dysregulation and poor sleep might promote AD pathogenesis through proinflammatory mechanisms and increased accumulation and deposition of Aβ. According to this model, bidirectional relationships exist between circadian dysregulation and poor sleep; inflammation and Aβ deposition; and AD pathogenesis and circadiandysregulation.

In this section the case is made for sleep quality as a critical determinant of AD risk and progression in older adults. The case is based on compelling evidence supporting the following assertions: (1) poor sleep quality is predictive of cognitive decline in older adults; (2) good sleep quality may protect against genetic susceptibility factors for AD, perhaps by increasing Aβ clearance from the brain; and (3) poor sleep quality is proinflammatory and associated with increased risk of developing metabolic and/or cardiovascular diseases, known to be independent risk factors for AD (see Figure 1 for a schematic outlining proposed pathways whereby circadian dysregulation and poor sleep are associated with AD pathogenesis). The importance of addressing sleep quality in the early stages of AD is emphasized not only from the patient’s perspective, but also as a means of reducing caregiver burden in an effort to delay the need for institutional care.

### Sleep in Alzheimer’s disease: impact on cognition and health

Sleep is vital for optimal health and well-being. Prolonged accumulation of sleep debt impairs physical, mental, and emotional fitness and can cause behavioral and mood disorders (Chokroverty and Avidan, 2012). The proinflammatory aspects of chronic sleep restriction are well documented (for reviews see Irwin, 2002; Faraut et al., 2012). Of particular importance to people at risk for AD, some sleep characteristics are predictive of cognitive decline in older adults, such as excessive daytime sleepiness, frequent awakenings, and nighttime sleep durations less than 6.5 h (Foley et al., 2001; Jaussent et al., 2012; Keage et al., 2012). Given how critical sleep is for cognitive function, it makes intuitive sense that sleep is an important factor in AD patient outcomes. However, the directionality of the relationship between sleep and AD has not always been clear. While it is generally accepted that sleep disruptions increase as a function of AD severity (Prinz et al., 1982a,b; Vitiello et al., 1990)—due at least in part to AD-related neuropathology of the circadian system (for review see Coogan et al., 2013)—the role disrupted sleep plays in AD onset and progression has yet to be fully appreciated.

Does disrupted sleep somehow cause or accelerate AD? Two recent studies have shed light on this issue: the first provides new evidence showing good sleep quality may be protective for individuals genetically susceptible to AD (Lim et al., 2013); while the second uncovers an underlying mechanism that might explain how sleep could protect against the formation of AD neuropathology (Xie et al., 2013). The first study, a 6 year longitudinal study of 698 community dwelling older adults without dementia, examined the relationship between sleep consolidation, cognitive function, AD incidence, and the development of Aβ and NFT pathology (as determined by post-mortem analysis of 201 participants who died during the study). Importantly, their analysis included comparisons for persons carrying the ε4- vs. ε4+ *APOE* genotypes. The results indicated better sleep consolidation (as measured by actigraphic sleep recordings comparing “good” vs. “poor” sleepers: 90th vs. 10th percentile, respectively) significantly attenuated the AD risk associated with the ε4+ genotype. Specifically, among ε4+ genotypes, better sleep consolidation was significantly associated with lower 6 year incidence rates of cognitive decline and NFT densities, but not Aβ pathology. In the second study, brain imaging of live mice was used to show interstitial space within the brain increases by as much as 60% during sleep, allowing for increased convective exchange of cerebrospinal fluid (CSF) with interstitial fluid (Xie et al., 2013). This increased CSF fluid exchange was also shown to significantly increase Aβ clearance during sleep. Taken together, these studies suggest a bidirectional relationship may exist between sleep and AD pathology, see Ju et al. (2014). As such, sleep disruptions characteristic of AD may involve a form of mutually destructive reinforcement whereby circadian-related sleep disruptions contribute directly to onset and progression of AD neuropathology, while at the same time AD neuropathology exacerbates circadian dysregulation of sleep-wake rhythms. In addition to these direct effects, poor sleep quality may increase AD risk indirectly as a moderator of other AD risk factors.

As mentioned earlier T2DM is an independent risk factor for AD and other dementias (Peila et al., 2002; Crane et al., 2013), particularly among individuals with the *APOE*-ε4 genotype (Peila et al., 2002). This increased risk is linked to cognitive deficits associated with impaired glycemic control in T2DM (Cukierman-Yaffe et al., 2009). Over the last decade, compelling evidence has identified disrupted sleep as an independent T2DM risk factor (Gottlieb et al., 2005; Knutson et al., 2006, 2007; Laposky et al., 2008; Gale et al., 2011; Touma and Pannain, 2011; Wan Mahmood et al., 2013), contributing to diabetes progression (Gale et al., 2011) and severity (Knutson et al., 2007). Poor sleep quality (as measured by the Pittsburgh Sleep Quality Index; PSQI) is predictive of higher levels of hemoglobin-A1c (HbA1c; Knutson et al., 2007; Wan Mahmood et al., 2013), which is the gold standard for indexing glycemic control and diabetes self-management (Canadian Diabetes Association Clinical Practice Guidelines Expert Committee, 2013).

Vascular disease is another well-known AD risk factor (Breteler, 2000; Kivipelto et al., 2006; Brown and Thore, 2011; Debette et al., 2011; Virta et al., 2013). In fact, some critics of the amyloid hypothesis have argued in favor of a “vascular hypothesis” as the genesis of AD (de la Torre, 2010; Marchesi, 2011; Drachman, 2014). This alternative hypothesis suggests that AD type dementia results from damage to small blood vessels, caused by oxidative-induced inflammation and dysregulated amyloid metabolism (Marchesi, 2011). As with T2DM, circadian dysregulation and resultant disruption of sleep-wake rhythms are independent risk factors for vascular disease (Brown et al., 2009; Eguchi et al., 2010; Ruiter Petrov et al., 2014) (for reviews, see Cappuccio et al., 2011; Palma et al., 2013). Thus in addition to the direct effects described earlier, circadian dysregulation and resultant sleep disruptions may increase AD risk indirectly as well by increasing one’s risk of developing T2DM and/or vascular disease. Consequently, for older adults at high AD risk—based on genetic susceptibility or other known risk factors—disrupted sleep should not simply be dismissed as a normal part of aging. Age-related changes in sleep are common; however, for individuals susceptible to AD, poor sleep quality may be a critical contributing factor increasing AD risk, progression and severity.

It is worth repeating here that a neuroinflammatory perspective of AD is compelling in that all of these well-established AD risk factors (poor sleep quality, T2DM, and vascular disease) have inflammation in common (see Finch, 2011; Watt, 2014). As such combating increased inflammation—as function of aging (i.e., “inflammaging”; Franceschi et al., 2007) and proinflammatory lifestyle factors (i.e., poor diet, limited exercise, and poor sleep quality; Watt, 2014)—is an intriguing target for future interventions to delay AD onset and progression.

### Sleep in Alzheimer’s disease: caregiver burden

Beyond the impact poor sleep may have on AD risk, addressing sleep quality in individuals with dementia is critical from the perspective of caregivers: Disrupted behavioral sleep-wake rhythms among AD patients typically result in sleep disruptions for their caregivers, which significantly increases caregiver burden and is a deciding factor in transitioning from community dwelling to institutional care. The majority of care for people with dementia is provided by family members (Prince and Jackson, 2009). Most family caregivers are women, typically wives of the demented and as such many are 65 years of age or older (Stone et al., 1987). In one study examining sleep disturbances in AD, 63% of spousal caregivers reported their sleep was disrupted by their demented spouse (Creese et al., 2008). A recent study has confirmed high prevalence of disrupted sleep among AD caregivers, though the rate was lower: 45% (Cupidi et al., 2012).

The sleep disturbances among caregivers are significantly more severe than the age-related sleep disruptions observed in age-matched controls (Wilcox and King, 1999). One study showed on average spousal caregivers sleep less than 6 h/night (McKibbin et al., 2005), which as discussed earlier is predictive of cognitive decline. In fact, there is evidence of cognitive deficits associated with caregiver status (i.e., AD spousal caregivers vs. age-matched non-caregivers) (Caswell et al., 2003). Caswell et al. (2003) showed that the variance in Digit Symbol Substitution test scores was explained by age, education, and caregiver status (i.e., AD caregivers scored significantly worse than non-caregivers).

In addition to cognitive impairment, sleep disruptions in AD caregivers is associated with poorer mental health (Willette-Murphy et al., 2006), decreased quality of life (QoL; Cupidi et al., 2012), and significantly increased caregiver burden (Allegri et al., 2006; Cupidi et al., 2012; Kamiya et al., 2014). The latter is of particular importance because caregiver burden is the strongest predictor of self-rated health in dementia caregivers (Abdollahpour et al., 2014). Most importantly—in terms of the economic impact of AD—disturbed sleep and its impact on burden of care is a primary trigger in the decision to transition from community dwelling to institutional care (Sanford, 1975; Pollak and Perlick, 1991; Gold et al., 1995). Findings from Sanford (1975) emphasized the importance of addressing sleep disruptions in AD: in their survey of family members who had recently placed their demented relative in an extended care facility, 84% said they would take their relative back if the sleep disturbances could be remedied.

To summarize briefly, addressing sleep quality in older adults at risk for AD is of profound importance because disrupted sleep is associated with: (1) increased AD risk through direct and indirect pathways; (2) increased AD severity both in terms of cognitive symptoms and neuropathology; (3) accelerated AD progression; (4) negative impacts on sleep, cognitive function, health, and QoL of AD caregivers; and as a result (5) dramatically increased caregiver burden—so much so that disrupted sleep is often cited as a primary trigger when families decide to institutionalize their demented relative. Therefore, the need to address sleep quality in AD patients and their caregivers is compelling. Whether or not sleep disturbances in AD can be addressed is, however, very much an unanswered question.

## Can we improve sleep in aging and Alzheimer’s disease?

Having characterized how sleep changes as a function of normal aging and AD, and given the severity of sleep disruptions associated with AD, the obvious questions that follow are: (1) Can these age-related changes be prevented in normal aging?; and (2) Can sleep quality—as defined both in terms of sleep duration and consolidation—be improved in older adults with AD? Put simply, the science to date is at best undecided; however there may yet be untapped potential in interventions aimed at improved sleep hygiene and enhanced circadian regulation. The following section discusses sleep hygiene and circadian regulation, exploring how they might provide the key to improving sleep in older adults. While interventions aimed at improving sleep might benefit older adults in general, we focus mainly on older adults with—or at high risk for—AD because of the particular importance sleep quality plays in the health outcomes of patients with AD. The sections that follow discuss a number of tools that may prove useful in addressing age-related sleep disruptions.

### Sleep hygiene

Sleep hygiene refers to a set of behavioral practices that can impact sleep quality, such that poor sleep hygiene could exacerbate or even cause poor sleep; and conversely, good sleep hygiene should result in feeling more rested and alert upon awakening, as well as a greater ability to function throughout the day—while experiencing less of the cognitive drag associated with excessive daytime sleepiness (Stepanski and Wyatt, 2003; Lockley and Foster, 2012; American Academy of Sleep Medicine, 2014). Poor sleep hygiene in AD patients is symptomatic of the disorder in the sense that they nap excessively during the day and get up often at night. Whereas a single nap during the afternoon—not exceeding 2 h—may be helpful both in terms of alleviating sleep debt and promoting cognition (Taub, 1979; Tamaki et al., 1999; Campbell et al., 2005), advocates of good sleep hygiene caution that excessive daytime napping likely worsens age-related sleep complaints (i.e., shortened duration and fragmentation of subsequent nighttime sleep; Hays et al., 1996; Stepanski and Wyatt, 2003; American Academy of Sleep Medicine, 2014).

The science to date is unclear on the degree to which efforts to improve sleep hygiene affect sleep quality and quantity in normal aging or AD. It may be that people with difficulty sleeping have already done what they are willing to do in terms of addressing their sleep hygiene. Or perhaps older adults have come to accept changes in their sleep as an inevitable part of aging, and thus have not considered the importance of good sleep hygiene. Regardless, given how critical getting quality sleep is for older adults at risk for dementia, further empirical studies are warranted to: (1) determine whether lifestyle interventions specifically designed for this population can improve sleep hygiene; and if so (2) determine whether better sleep hygiene translates to increased quality of sleep.

### Effects of light on circadian rhythms and sleep

In comparison to the effects of sleep hygiene on sleep quality, the impact of light on circadian regulation and sleep-wake rhythms is much better understood. Light is the primary time cue our biological clock uses to entrain physiology and behavior with the solar day-night cycle. As such, exposure to light (even low-level light from lamps and electronic devices) can change the timing of circadian rhythms. These changes in circadian timing are called phase shifts. Much like we can adjust our watches forward or backward when traveling to a new time zone; light can shift our biological clock forward (i.e., phase advance) or backward (i.e., phase delay). The effect of light on circadian rhythms is time-dependent, meaning the magnitude and direction of phase shifts depend on the timing of light exposure relative to T_b_-min. A phase response curve (PRC) is a graphical representation of stimulus effect (i.e., magnitude of the phase shift) plotted as a function of the time the stimulus is applied, typically in relation to T_b_-min.

The time-dependent phase shifting effects of light have been well characterized elsewhere (Czeisler et al., 1989; Khalsa et al., 2003; Revell et al., 2012; St Hilaire et al., 2012; Rüger et al., 2013). In summary, phase delays result from exposure to light at night before T_b_-min—when we would normally be sleeping, well after the time our biological clock is expecting light. Conversely, circadian rhythms are phase advanced by early morning light exposure following T_b_-min. Thus, in terms of human circadian sleep-wake rhythms: (1) light at night before T_b_-min delays the rhythm, resulting in a later sleep-onset and a later wake-onset the following morning (assuming the absence of other factors such as an alarm clock); and (2) early morning light shortly after T_b_-min advances the rhythm such that subsequent sleep and wake onsets occur earlier than they did prior to the light exposure. The magnitude of a light-induced phase shift is greatest in the hours immediately prior to T_b_-min (delays) and following T_b_-min (advances) (Dawson et al., 1993).

In addition to time-dependence, several factors influence the magnitude of light-induced phase shifts including light duration, intensity, and color (Khalsa et al., 2003; Revell et al., 2012; St Hilaire et al., 2012; Rüger et al., 2013). In general, magnitude increases with increased duration and/or intensity, and full-spectrum “white” light works best; however, short-wavelength “blue” light (450–500 nm) has been shown to be nearly as effective as “white” light (Revell et al., 2012; Rüger et al., 2013). Bright light—when used in accordance with the principles of the known PRC to light—is highly effective at synchronizing circadian rhythms of physiology and behavior. Beyond its role in synchronizing circadian rhythms, bright light has well defined “alerting” effects that can combat sleepiness, elevate mood, and improve cognitive function (Badia et al., 1991; Cajochen et al., 2000; Plitnick et al., 2010; Münch et al., 2012).

To date, bright light has been used successfully as a non-pharmacological therapy to address a number of circadian regulation related disorders including: (1) delayed sleep phase onset in young adults (Gradisar et al., 2011; Saxvig et al., 2014); (2) seasonal affective disorder (Glickman et al., 2006; Rastad et al., 2008); (3) shiftwork induced internal desynchrony (Crowley et al., 2003; Smith et al., 2009); (4) jet-lag (Forbes-Robertson et al., 2012; Weingarten and Collop, 2013); and even (5) to address sleep disruptions in nursing home residents (Kobayashi et al., 2001; Fetveit et al., 2003; Alessi et al., 2005)—though there is considerable variability in results with this population (as will be discussed in detail later).

### Effects of melatonin on circadian rhythms and sleep

Melatonin is a pineal gland produced hormone with modest chronobiotic properties, though weak in comparison to circadian phase-shifting effects of light (Arendt and Skene, 2005). However the PRC to melatonin is opposite that of light, such that oral administration at night—before T_b_-min—causes advances; and melatonin in the morning—after T_b_-min—delays circadian rhythms (Lewy et al., 1998). The timing of pineal melatonin production is controlled by the SCN—with low levels produced during the day, rising levels ~2 h before habitual sleep-onset, and peak levels at night in advance of T_b_-min (Arendt and Skene, 2005; Burgess and Fogg, 2008). Melatonin is thought to be involved in initiating sleep-onset because: (1) timing of the melatonin rise coincides with opening of the circadian sleep gate; (2) this rise in melatonin level is associated with increased sleepiness and lowered core body temperature; (3) oral melatonin administration during the day has soporific effects; and (4) disrupted sleep at night is sometimes associated with decreased amplitude of melatonin during the sleep phase (Dijk and Cajochen, 1997; Arendt and Skene, 2005; Pandi-Perumal et al., 2005).

Given its chronobiotic properties, melatonin is often used in combination with bright light to address circadian regulation related disorders (Crowley et al., 2003; Saxvig et al., 2014). Melatonin is particularly useful as a chronobiotic for blind people who lack the ability to use light to synchronize their physiology and behavior to a desired work/social schedule (Skene and Arendt, 2007). When taken daily—at an appropriate time—oral melatonin administration in blind people reverses circadian desynchrony, improves nighttime sleep, and reduces daytime napping (Skene and Arendt, 2007).

### Effects of exercise on circadian rhythms and sleep

The chronobiotic properties of exercise have been well established in various animal models (typically using wheel running in rodents; for reviews see Mrosovsky, 1996; Hastings et al., 1998). Whether or not exercise has chronobiotic properties in humans is difficult to determine given the challenges associated with isolating the effect of exercise vs. other factors such as light, food, and social influences (for reviews see Mistlberger and Skene, 2004, 2005). However studies in blind people who lack sensitivity to light—but remain able to entrain to daily work/social schedules without using exogenous melatonin—suggest that non-photic stimuli are capable of synchronizing circadian rhythms (reviewed in Mistlberger and Skene, 2005). Whether it is exercise, social influences, food (i.e., regularly scheduled mealtimes) or a combination of all of these that provides the critical entrainment signal is yet to be defined empirically.

Nonetheless, sufficient data exists from studies examining the phase-shifting effects of exercise to produce an exercise/non-photic PRC in humans (Mistlberger and Skene, 2005). In general the results indicate that: (1) exercise in the evening before habitual sleep-onset advances circadian rhythms; (2) exercise during the habitual sleep time delays circadian rhythms; (3) the exercise PRC in humans is consistent with ones reported in animal models; (4) the PRC to exercise is sufficiently different from that of light to diminish concerns of light as a confound; and (5) in humans the phase-shifting effects of exercise are significant but modest in magnitude, when compared to light or melatonin. The effects of exercise on sleep quality, however, may be of greater consequence.

Exercise has long been thought to play a role in the quality of sleep. Epidemiological studies examining the relationship between exercise and sleep (see reviews Driver and Taylor, 2000; Youngstedt, 2005) consistently show that: (1) when asked an open ended question about behaviors that promote better sleep, exercise is listed as the most important; and (2) based on self-report data, people who exercise more also report having better quality sleep and reduced daytime sleepiness, compared with individuals who are more sedentary. These studies can only reveal relationships, of course, and countless variables could explain the correlation between exercise and sleep quality, including general health and well-being and many other life-style factors. As such, controlled experiments are important to determine the effects of acute and chronic exercise on sleep (Driver and Taylor, 2000; Youngstedt, 2005).

A meta-analytic examination of the empirical literature on acute exercise and sleep (Youngstedt et al., 1997) suggests that: (1) acute exercise increases total sleep time (TST) and alters sleep architecture (i.e., increased slow wave sleep; SWS; and decreased REM sleep); (2) exercise increases REM-onset latency, which may explain the decrease in REM sleep duration (i.e., an exercise-induced increase in SWS may delay REM-onset and thereby decrease REM duration)—note that perhaps a REM rebound would be seen in subsequent sleep bouts as has been shown during multi-night electroencephalogram (EEG) recordings of recovery sleep following sleep deprivation (reviewed in Bonnet, 2000); (3) exercise improves sleep consolidation as determined by decreased wake time after sleep-onset (WASO: time spent awake after sleep onset, a measure of sleep fragmentation—the inverse of sleep consolidation); (4) duration of exercise moderates the effect of exercise on sleep, with effects requiring durations of at least an hour; and (5) the magnitude of exercise effects on sleep are modest and time dependent, with greatest effects seen when exercise occurs 4–8 h prior to sleep-onset. Note however, that the majority of studies have been conducted on young, athletic, “good” sleepers—raising the possibility that the modest effects seen are the result of ceiling effects.

Another potential explanation for the modest effect sizes observed could be a simple function of the limited number of electrodes typically used for EEG recordings of sleep. Interestingly, a separate study showed location dependent increases in SWS following a motor adaptation task (Huber et al., 2004). This study used a 256-channel system for EEG recordings, providing much greater spatial resolution. Their results indicate increases in SWS were focused around the brain area involved in the motor-adaptation task. Admittedly, 256-channel EEG recordings are impractical for regular use in sleep studies as they make sleep uncomfortable. Our point is simply that failure to detect large effects of exercise on EEG sleep recordings may in part be a function of the measurement tools employed. Thus it remains possible that exercise could have greater effect in older adults who tend to be “poor” sleepers, and that these effects may exist beyond our ability to detect important changes. Indeed—with respect to the former—studies in older populations provide evidence supporting age as a moderator of exercise effects on sleep (Driver and Taylor, 2000); but there have been too few studies to date in older subjects, and the data they provide typically relies on self-report measures of sleep.

In recent years, however, exercise in older adults has become the focus of important research on a separate, but related topic. Exercise—as a promoter of cognitive function and neural plasticity—has become a promising line of inquiry both in normal aging (Colcombe and Kramer, 2003; Colcombe et al., 2004; Liu-Ambrose et al., 2010, 2012); and as an intervention for older adults at risk for AD (Lautenschlager et al., 2008; Baker et al., 2010; Nagamatsu et al., 2012, 2013). We mention these studies in this section because sleep may play a role as mediator or moderator of exercise-effects on cognitive function in older adults. The impact of sleep on cognitive performance is well known. Given the effect exercise has on sleep, it makes intuitive sense that improved sleep—following exercise—could be a factor in observed exercise effects on cognitive performance. Interestingly, findings from a recent study suggest physical activity can moderate the effects of poor sleep on executive function in older adult women (Lambiase et al., 2014). Thus further study is warranted to explore the mechanisms by which exercise, sleep, and cognitive function are related—specifically in older adults. In particular, studies are needed to explore potential synergistic effects of interventions that combine exercise and improved sleep quality.

### Effects of food on circadian rhythms

Food has potent chronobiotic properties in animal models (for reviews see Mistlberger, 1994; Stephan, 2002). However it is not yet clear that food has the same effect on human circadian rhythms; mainly because few properly controlled studies have examined food-entrainment in humans (reviewed in Mistlberger and Skene, 2005); and perhaps because for humans eating has become somewhat of a social activity and we tend to eat more for pleasure than to serve a metabolic need. Importantly, entrainment to mealtime is SCN independent (i.e., SCN-lesioned animals can synchronize circadian rhythms to a restricted feeding window; Mistlberger, 1994; Stephan, 2002). While the properties of food-entrainment have been well characterized, efforts to identify the neural substrate of this SCN-independent mechanism have not succeeded—despite decades of research and countless lesion studies—suggesting that the chronobiotic properties of mealtime are very robust and likely rely on a distributed system of multiple redundant structures (Landry, 2013). A distributed system able to withstand neural insults and injury makes intuitive sense, given the survival importance of capacity to anticipate food availability in a cyclical environment—at or near the top in terms of the hierarchy of needs. Because SCN-atrophy is characteristic of AD, regularly scheduled mealtimes might provide an SCN-independent approach to enhance circadian regulation in older adults with AD.

Indeed, food-entrainment as a means of enhancing circadian regulation in AD is the subject of an interesting review (Kent, 2014). Note that while mealtimes in nursing homes are likely already regularly scheduled, the amount of actual food intake that occurs during these set mealtimes is likely highly variable. Thus the use of food as a signal to enhance circadian regulation in AD remains an intriguing possibility and well worthy of investigation for future studies. Particularly given evidence establishing calorie restriction as an effective anti-aging intervention (see Watt, 2014). Further support for examining food-entrainment as an AD intervention comes from studies examining the adaptive significance of *APOE* genotype. The distribution of *APOE* alleles differs geographically (Singh et al., 2006), linked in some way to diet and food availability (Corbo and Scacchi, 1999; Egert et al., 2012): ε3 is more prevalent in populations with long-established agricultural economies; whereas ε4 is most prevalent in areas where foraging for food is still dominant and supply is limited (Corbo and Scacchi, 1999). Evidence from studies in mice and humans suggests the ε3 allele confers a selective advantage when food supply is abundant—particularly for diets high in fat—while ε4 may be more adaptive when food is scarce, due to its increased efficiency in cholesterol extraction from foods (Corbo and Scacchi, 1999; Egert et al., 2012). The transition from foraging to an agricultural economy and increased longevity may explain the shift in the population distribution of the *APOE* genotype from ε4—considered the ancestral genotype (Egert et al., 2012)—to predominantly ε3. Currently ~90% of the general population is heterozygous-ε3 and ~60% are homozygous-ε3/ε3 (Roses, 1996).

Perhaps the increased AD risk associated with the ε4 allele is reflective of evolutionary discordance (Konner, 2001), resulting from a large divergence between the current proinflammatory diets and lifestyles of Western societies and the original evolutionary environment in which our genetic makeup was forged (see Watt, 2014 for more on this topic). If so, food restriction may prove to be an important tool for improved circadian regulation and combating inflammation in AD and other diseases of aging.

## The rationale and science to date for chronotherapy in older adults with Alzheimer’s disease

Given the nature and severity of sleep disruptions in AD, the potential for disrupted sleep to exacerbate cognitive decline, and its impact on caregiver burden; there is urgent need for interventions capable of improving sleep quality among older adults with AD. Because sleep disruptions increase as a function of AD severity—the result of AD-associated pathological changes in the SCN—AD patients in residential care facilities tend to have very poor circadian sleep-wake rhythms. These disruptions in circadian regulation are probably exacerbated by the sedentary lifestyle typical of residential care facilities—which leads to very limited exposure to sunlight or any other form of bright light (Campbell et al., 1988; Shochat et al., 2000). Thus, AD patients in residential care are a particularly important target for sleep interventions. Chronotherapy aims to enhance circadian regulation of sleep-wake rhythms using appropriately timed exposure to zeitgebers (i.e., German for “time giver”: time cues capable of entraining circadian rhythms; e.g., light, melatonin, exercise, and food). Because light is the most potent zeitgeber, it may provide the best option for a chronotherapeutic intervention designed to enhance circadian regulation and improve sleep quality of AD patients in nursing homes. Bright light therapy (i.e., application of full-spectrum bright light, specifically timed in accordance with the human PRC to light; BLT) has been shown to improve circadian regulation and sleep in community dwelling older adults (Kohsaka et al., 1998; Kobayashi et al., 1999) and older adults in nursing homes (Kobayashi et al., 2001; Fetveit et al., 2003), leading to speculation the same may be true for older adults with AD. However, these reports were based on preliminary studies using very limited participant numbers and the effects were modest. Nonetheless randomized controlled trials (RCTs) examining the efficacy of BLT to improve circadian regulation and sleep in older adults with AD were warranted.

### Bright light therapy in older adults with dementia: randomized controlled trials

Despite promising preliminary data early on from non-randomized studies (Mishima et al., 1994; Fetveit et al., 2003) and RCTs (Mishima et al., 1998; Lyketsos et al., 1999; Ancoli-Israel et al., 2002), two Cochrane meta-analytic reviews of RCTs testing light therapy in older adults with dementia have yielded disappointing results (Forbes et al., 2009, 2014). The Cochrane reviews included all RCTs of light therapy at any intensity and duration testing effects on cognition, ADLs, sleep, challenging behavior, and psychiatric symptoms associated with dementia of any type. The updated Cochrane review published in 2014 examined 13 articles from 11 RCTs with a combined total of 499 participants and a drop-out rate of ~20%. Their results indicate light therapy had no significant effect on any of the outcome measures, except for ADLs: a single RCT examined the effect of light therapy on AD-related progressive decline of ADLs (Riemersma-van der Lek et al., 2008)—this longitudinal study (up to 3.5 years) reported whole-day bright light (~1000 lux) vs. dim light (~300 lux) resulted in significant attenuation of the gradual increase in functional limitations—a 53% reduction in the slope of ADL limitations. Admittedly reductions in the progression of ADLs is an important finding; but if light is such a potent chronobiotic, then why are RCTs of light therapy yielding such uninspiring results for other outcome measures? A close examination of the methods employed by these studies reveals a number of important factors that could explain the null findings reported in the 2014 Cochrane review.

#### Limited sample size

Beyond the impact of having so few studies that met the inclusion criteria for the Cochrane reviews (11 RCTs in total), the limited number of participants recruited for the majority of these RCTs probably hindered power to detect effects of light: 6/11 RCTs had fewer than 34 participants combined across all conditions (Mishima et al., 1998; Lyketsos et al., 1999; Graf et al., 2001; Fontana Gasio et al., 2003; Dowling et al., 2008; Nowak, 2008); and of the five remaining studies, only one had more than 34 participants assigned to an active light condition (Riemersma-van der Lek et al., 2008).

#### Unknown circadian phase of Bright Light Therapy administration

The effect of light on circadian rhythms is time-dependent, with greatest effect known to occur in the hours immediately surrounding the T_b_-min. For this reason, all of the studies that defined the human photic PRC did so by timing light exposure in relation to assessed circadian phase of the SCN—as determined by T_b_-min or measuring each participants dim light melatonin onset (DLMO: a standard measure of circadian phase assessed by determining the onset of increased melatonin secretion—assayed in saliva or blood—under dim light conditions because light exposure suppress melatonin secretion (Pandi-Perumal et al., 2007)). For practical reasons, all of the RCTs included in the Cochrane review timed BLT in relation to clock time (i.e., a set time each morning, afternoon, or evening) rather than by assessed circadian phase of the participants. Given that circadian dysregulation is symptomatic of dementia, it is very likely that circadian phase varied significantly across participants. Therefore it is impossible to know what circadian phase BLT was administered—which is critical if one intends to address circadian dysregulation using BLT. As such, failure to time BLT in relation to circadian phase would dramatically increase variability of the outcome measures and thereby hinder power to detect effects of light.

#### Insufficient light intensity

As is so for time-dependence, light intensity is a critical factor determining the magnitude of effects on circadian regulation. With two exceptions (Fontana Gasio et al., 2003; Riemersma-van der Lek et al., 2008), all of the Cochrane RCTs used light intensities that have been sufficient to shift circadian rhythms in previous studies; however the vast majority of these studies (8/11) used light intensities at the mid-low range of known efficacy 5000 lux: (Mishima et al., 1998); 3000 lux: (Graf et al., 2001); 2500 lux: (Ancoli-Israel et al., 2003; Dowling et al., 2005, 2008; McCurry et al., 2011); 1000 lux:(Riemersma-van der Lek et al., 2008); and a dawn-dusk simulator using ~200 lux: (Fontana Gasio et al., 2003). The intensity threshold for BLT-efficacy on circadian regulation was tested primarily in healthy young adults; however light perception varies significantly with age (note that the average age of participants in the Cochrane RCTs was ~80). Furthermore, evidence suggests sleep deprivation can attenuate light-induced phase shifts—at least in an animal model (Mistlberger et al., 1997)—and as discussed earlier sleep disruptions are common in older adults. Therefore BLT in older adults, using lower light intensities (i.e., <3000 lux) probably increases variability in results, further diluting power to detect effects of light. In older adults—to combat decreased light perception and attenuated photic shifting—we suggest using brighter light intensity with blue-enriched spectrum is best practice.

#### Insufficient light duration

We can apply a similar argument with respect to the typical duration of BLT exposure used in the Cochrane RCTs. With only one exception (Riemersma-van der Lek et al., 2008), all of the Cochrane RCTs used 2 h or less of BLT exposure, which may work perfectly well in young adults with intact circadian regulation, but is likely significantly less effective in demented older adults with compromised circadian systems.

#### Insufficient baseline data

With respect to actigraphic measures used to provide estimates of sleep parameters—because of the highly variable nature of recordings in demented elderly which vary not only from night to night but also week to week—baseline recordings of at least 14-days are recommended (Van Someren, 2007). Unfortunately, the vast majority of the Cochrane RCTs (9/11) used baseline recordings of only 7-days or less, which has been shown insufficient to provide acceptable reliability when estimating sleep parameters in elderly demented (Van Someren, 2007).

#### Insufficient intervention duration

Given the advanced average age of participants (~80 years)—and the likelihood that for many of the participants circadian dysregulation had progressed over the course of more than a decade—it would seem plausible that reversing the process would also take considerable time; and yet only one of the Cochrane RCTs employed a longitudinal design using BLT for more than 6 months (Riemersma-van der Lek et al., 2008). Interestingly, this is the study that reported a greater than 50% decline in the rate of increased functional deficit in ADLs during BLT compared to dim light controls. Furthermore, the authors reported progressive improvements in sleep duration for the BLT condition, which continued for the duration of the intervention (up to 3.5 years).

### Bright Light Therapy in older adults with dementia: further study is warranted

Our intention in the previous section is not to cast aspersions on what we believe to be important studies that aimed to define the utility of BLT for a population in desperate need of intervention. We hope only to place in proper perspective the findings from the current Cochrane review. While we agree with the authors’ conclusion that there is an absence of evidence in favor of BLT for use in elderly demented, there are sufficient methodological shortcomings in these studies for us to urge caution when interpreting their findings: absence of evidence is not evidence of absence! This line of scientific inquiry is no doubt in its infancy, and thus, we argue further study—using improved methods with greater power to detect effects—is imperative.

That being said, we acknowledge the possibility that among demented elderly, decades of progressive decline in circadian regulation may have rendered the system unresponsive to a singular approach. Furthermore, dementia is a heterogeneous disease with multiple etiologies, and thus, one-size-fits-all interventions are likely doomed to fail. Combined interventions using all known chronobiotics (i.e., light, melatonin, exercise, and food) may be required to rescue circadian regulation in elderly demented; and these combined interventions may only be effective for specific types of dementia. Perhaps an even better approach is to intervene before neurodegeneration has taken its toll on the circadian system. Can we identify individuals at high risk for dementia before the disease takes hold? Is there a prodromal stage of AD during which circadian regulation can be preserved?

## Mild cognitive impairment

Identification of risk factors, combined with early diagnosis and intervention, is best practice for any disease or disorder. However as detailed in this review—for individuals with cognitive decline on a trajectory toward dementia—early intervention may be even more critical, at least with respect to preserving the quality of their sleep. Sleep changes as a function of normal aging, but in this population disrupted sleep may be particularly detrimental. Chronotherapy—targeting improved regulation of circadian sleep-wake rhythms—will likely have greatest effect in the early stages of dementia’s development; before neuropathology of the circadian system has progressed to a point that renders the SCN less responsive to entrainment cues. Fortunately over the last two decades, early detection of a prodromal stage of dementia has been the focus of intensive study. In the late 80’s, clinicians and researchers identified a state of cognitive deficit that existed between normal aging and pathological decline (Reisberg et al., 1988). Soon after, the theoretical construct “Mild Cognitive Impairment” (MCI) was introduced as a clinical entity to capture this gray zone of cognitive impairment—beyond what is expected in normal aging but not severe enough to satisfy criteria for dementia diagnosis (Petersen et al., 1999). Since then MCI has evolved as a clinical entity. Its predictive utility for identifying individuals with cognitive deficits that will later convert to dementia diagnosis has become the subject of great debate and controversy (for a detailed review of MCI and its evolution, see Petersen et al., 2014). Here we provide a brief overview of the state of the MCI science to date.

### Mild cognitive impairment: definition and classification

Much like dementia with its diverse etiologies, MCI is a heterogeneous entity whose definition has been refined several times over the last 20 years. As a diagnostic entity MCI is broadly defined as cognitive impairment—based on a subjective complaint subsequently confirmed by objective cognitive measures—not explained by normal aging and not severe enough for dementia diagnosis (Petersen et al., 2014). Preserved independence in functional abilities (as measured by ADLs) is a key criterion that distinguishes MCI from dementia. Cognitive impairment is defined as poor performance in one or more of the following domains: executive functions, attention, language, memory, and visuospatial skills. Mild cognitive impairment is classified by domain of impairment beginning with the memory domain: amnestic MCI (a-MCI) when memory is impaired; and non-Amnestic MCI (na-MCI) when memory is not impaired. Mild cognitive impairment is further classified by number of domains impaired (i.e., single vs. multiple domain MCI) (Petersen et al., 2014).

### Mild cognitive impairment: prevalence, risk factors, neuropathology and neuroimaging

Using the current definitions—averaged across the major population-based studies—worldwide prevalence of MCI and a-MCI among adults over 65 years is 18.9% (Petersen et al., 2014) and 7% (Ward et al., 2012a), respectively. That being said, prevalence estimates vary greatly across studies highlighting the need for enhanced standardization of operational definitions of MCI and its various subtypes (Ward et al., 2012a). With respect to risk factors, MCI is much the same as dementia: age, education, *APOE* genotype, vascular disease, and diabetes have all been identified (reviewed in Petersen et al., 2014). Results for MCI studies vary between cross-sectional and prospective studies; and as seen with risk factors associated with cognitive decline in dementia—when compared with prospective studies—cross-sectional studies consistently report stronger associations for comorbidities such as vascular disease and diabetes (Petersen et al., 2014). Whereas MCI can be defined on a continuum of cognitive impairment distinct from normal aging and dementia; efforts to define a profile of MCI-specific neuropathology have been unsuccessful. A recent systematic review (Stephan et al., 2012) examining 162 studies of MCI-associated neuropathology concluded that MCI is heterogeneous and neuropathologically complex, making identification of a definitive MCI profile problematic without systematic improvements in the science including: representative brain tissue banks using standardized MCI criteria; and standardized neuropathological protocols. Nonetheless, numerous pathological changes associated with MCI have been identified including: (1) Aβ plaque and NFT formations; (2) vascular pathologies; (3) neurochemical deficits; (4) cellular injury; (5) inflammation; (6) oxidative stress; (7) mitochondrial changes; (8) changes in genomic activity; (9) synaptic dysfunction; (10) disturbed protein metabolism; and (11) disrupted metabolic homeostasis (Stephan et al., 2012). Obviously neuropathology makes MCI distinct from normal aging, but at present there is no clear demarcation in neuropathology that defines the conversion from MCI to dementia. However—in terms of identifying stages of decline—there may be hope for the future given that at least preliminarily it seems neuropathology associated with a-MCI may be more informative than other MCI subtypes (Petersen et al., 2006; Stephan et al., 2012).

### Mild cognitive impairment: circadian rhythms and sleep

As MCI research progresses and refinements are made in identifying cohorts at greatest risk of near-term cognitive decline, we will enhance our ability to target individuals who could benefit most from interventions designed to promote—or at least preserve—cognitive function. Because improved regulation of circadian rhythms and sleep is critical in older adults with AD, characterizing these parameters in older adults with MCI should be a priority. Unfortunately, relative to the number of studies examining sleep in normal aging and AD, there is a paucity of studies characterizing sleep in MCI. However two studies have provided valuable insight into the nature of circadian regulation of sleep-wake rhythms in MCI (Naismith et al., 2014) and how changes in circadian regulation may contribute to MCI and its conversion to AD (Tranah et al., 2011). Naismith et al. (2014) is the first MCI study to our knowledge that combined 14 day sleep-wake actigraphy and polysomnographic (PSG) assessments with melatonin sampling. Admittedly sample size was limited (30 MCI vs. 28 age-matched controls), but their results indicate significant differences associated with MCI: (1) the DLMO is phase advanced in MCI compared to controls; (2) habitual sleep-onset is significantly earlier in MCI vs. controls; (3) sleep disruptions (defined by WASO using actigraphy) are significantly higher in MCI vs. controls; and REM onset occurs later in MCI vs. controls. These findings suggest circadian rhythms and sleep in MCI are significantly different from age-matched healthy controls, in a manner similar to the changes observed in AD.

Interestingly, these MCI associated changes in melatonin onset and habitual sleep-onset are consistent with disruption of the circadian gated wake-maintenance zone discussed earlier. The second noteworthy MCI study (Tranah et al., 2011) provides preliminary evidence that decreased amplitude and robustness of the circadian sleep-wake rhythm precedes MCI and is associated with increased risk of MCI and conversion to dementia. The study used actigraphy to assess baseline circadian sleep-wake rhythms in 1282 healthy community-dwelling women whose average age was 83 years. Cognitive function was assessed at baseline using the MMSE and again with more comprehensive measures at follow-up almost 5 years later, on average. Their findings—circadian dysregulation preceded MCI and increased risk of AD conversion—are very intriguing; so much so that they were highlighted for review in “Critically Appraised Topics” (Schlosser Covell et al., 2012). Regrettably this study has significant methodological limitations, as discussed in Schlosser Covell et al. (2012): (1) participants with baseline MMSE score >24 were deemed cognitively normal, but scores of 24–27 can meet criteria for MCI, which may have led to overestimating conversion rates; and (2) only 3 days of actigraphy were used to assess circadian sleep-wake rhythms, which is insufficient given the variability inherent in actigraphic recordings. As such, further studies are required to explore the relationship between circadian regulation of sleep-wake rhythms, MCI, and its conversion to AD.

Mild cognitive impairment and dementia are heterogeneous and thus it makes intuitive sense that associated sleep disruptions—and the benefits of sleep interventions—would vary as a function of etiology. Presumably, sleep interventions would have greatest effect in individuals most likely to develop AD-related neuropathology of the circadian system, assuming intervention is possible early in the disease process. As such, MCI may be useful to identify individuals who are at higher risk of developing dementia—by age-matched comparison; however, scarce resources would be better utilized if we could stratify risk of conversion from MCI to AD-type dementia. The National Institute on Aging—Alzheimer’s Association (NIA-AA) has proposed criteria to define an AD spectrum within MCI using specific biomarkers associated with AD (i.e., valid indicators of Aβ deposition and neuronal injury; Albert et al., 2011). We suggest research priority should focus on identification, testing, refinement, and implementation of chronotherapeutic interventions—targeting older adults with MCI—with an emphasis on individuals most likely to convert to AD-type dementia (i.e., individuals with a-MCI and AD associated biomarkers/genotype).

## Conclusion

Without discovery of a cure or interventions that delay disease progression, dementia’s annual worldwide economic impact is expected to surpass $1 trillion USD as early as 2030. A cure for dementia—if one is possible—is likely many years away. Thus, a research imperative is to reduce dementia’s economic impact through interventions that promote cognitive function, preserve capacity for independent living, and delay need for institutional care. We argue that disrupted circadian sleep-wake rhythms in AD are of particular importance, given their impact on cognitive function. Sleep consolidation and duration change as a function of normal aging. However in AD—by comparison—disruptions of circadian sleep-wake rhythms are exaggerated. These disruptions become extreme as AD progresses from moderate to severe. In addition to their potential to accelerate cognitive decline, AD-related sleep disruptions greatly increase caregiver burden; which is a primary trigger in the decision to transition from community dwelling to institutional care. By improving sleep in AD, chronotherapeutic interventions might reduce the burden of AD—thereby buying time for the discovery of a cure. Because light is the most potent chronobiotic, BLT may have the greatest potential for use in AD. Alas, results of RCTs using BLT have been highly variable and effects are at best modest. However, to date, there have been too few RCTs to draw definitive conclusions. Furthermore, we discussed several methodological issues that could explain the disappointing results so far. As such, we believe the science examining chronotherapy for use in AD remains in its infancy.

### Where we need to go: a road map for future trials

#### Defining populations at high Alzheimer’s disease-risk

By itself MCI may be far from ideal as a near-term indicator of conversion to AD—lacking sensitivity and specificity as operationally defined to date—however, when used in combination with biomarkers of Aβ deposition and neuronal injury there may be greater potential to stratify AD risk. If future RCTs are to accurately assess MCI interventions, it is imperative that studies recognize this heterogeneity by employing stratified designs that identify an intervention’s differential effects across the NIA-AA’s *Uncertain*, *Intermediate*, and *High* classifications for probability of MCI due to AD. Furthermore, under this MCI umbrella—in combination with biomarkers that differentiate AD risk—key “at risk” sub-populations warrant research priority in future RCTs: (1) individuals with the *APOE*-ε4 genotype; and (2) individuals with comorbid conditions including diabetes, cardiovascular disease, and stroke. Presumably, regulation of circadian rhythms and sleep will be differentially impaired across these sub-populations, and thus, chronotherapy should be assessed for each sub-population independently.

#### Chronotherapy to protect sleep and preserve cognitive function

##### Bright Light Therapy—brighter, bluer, and for longer

To date, there have been too few RCTs testing BLT in older adults with dementia to come to any conclusions regarding its potential to improve sleep quality in this important population. The duration and intensity of BLT typically employed by these RCT’s—while shown to be effective in healthy young adults—may not have been sufficient to enhance circadian regulation for older adults, perhaps due to age-related changes in light perception and within the SCN itself. We recommend testing brighter light intensities for longer durations, and where possible blue-enriched light should be used. Furthermore, BLT needs to be tested in community dwelling MCI populations to determine if early intervention would have greater efficacy than has been shown previously in institutionalized AD patients.

With regard to timing of BLT, we suggest RCTs are needed to independently assess three distinct approaches: (1) early morning light; (2) all day bright light; and (3) evening light. *Early morning light* for as long as is practical should promote enhanced circadian regulation by providing a strong, consistent entrainment signal for an aging biological clock. Note that avoiding nighttime light is equally important in order to avoid conflicting signals. As such—for BLT to be effective—the priority is to hit the biological clock with a strong, unambiguous demarcation between day and night.

Increasingly limited exposure to bright light during the day is a consequence of the relatively sedentary lifestyle typical of advanced age—decreased daytime light exposure is particularly problematic in nursing homes (Campbell et al., 1988; Shochat et al., 2000). This decreased exposure to daytime light may contribute to age-related amplitude suppression of melatonin secretion at night: bright light during the day has been shown to increase melatonin amplitude at night (Park and Tokura, 1999); whereas dim light (i.e., <200 lux) at night dramatically suppresses melatonin secretion (Gooley et al., 2011). Thus, age-related decreased melatonin amplitude could be related to lower daytime light exposure and more nocturnal light exposure (e.g., getting up to go to the bathroom; watching TV or reading late at night when one cannot sleep).

Melatonin amplitude at night is correlated with sleep quality and duration (Morris et al., 1990; Pandi-Perumal et al., 2005), so decreased nocturnal melatonin might be a contributing factor in age-related sleep disruptions. Therefore, RCTs testing *all day bright light*, as used in Riemersma-van der Lek et al. (2008)—but with brighter, blue-enriched light, aimed at increasing melatonin amplitude and increasing sleep duration—would be informative. Finally, *evening light* (i.e., BLT coordinated with the beginning of the wake-maintenance zone, but well before habitual sleep onset) may be useful in two ways: first, it might enhance functioning of the wake-maintenance zone; and secondly, it may help extend sleep duration by delaying T_b_-min.

##### Melatonin—at bedtime in combination with Bright Light Therapy

In addition to its well characterized chronobiotic properties (Arendt and Skene, 2005), melatonin has recently become a research priority for its anti-oxidant properties including reduced inflammation due to oxidative stress (Reiter et al., 2000) and its potential for combating cancer progression (Margheri et al., 2012; Sánchez-Hidalgo et al., 2012). As such—if possible—reversing age-related reduced amplitude of nocturnal melatonin secretion (Burgess and Fogg, 2008) may be important for improving not only sleep, but general health in older adults as well. Melatonin supplements (2.5 mg) taken orally at night—by itself or in combination with all day BLT—can improve sleep in nursing home residents; but by itself negative side-effects on affect and behavior were observed (Riemersma-van der Lek et al., 2008). Therefore—at least when used in older adults with AD—low-dose melatonin should be used in combination with BLT. Furthermore, the long-term effects of melatonin supplements have yet to be determined, and thus, additional study is warranted. As an alternative, dietary sources such as tart cherry juice have recently been shown to increase nocturnal melatonin levels and enhance sleep quality (Howatson et al., 2012).

##### Exercise and social engagement—is anytime a good time?

Exercise is an effective intervention that promotes cognitive function and neural plasticity in MCI populations (Lautenschlager et al., 2008; Baker et al., 2010; Nagamatsu et al., 2012, 2013); however, the mechanisms involved have yet to be explained. Exercise has long been associated with better sleep—and given the importance of sleep for cognitive function—it remains possible that improved sleep is an underlying mechanism by which exercise promotes cognitive function in MCI. Future RCTs of exercise effects on cognitive function that also include quantitative measures of sleep (e.g., actigraphy or EEG) would be informative. Given that exercise trials to date have typically used exercise sessions 2–3 days/week, we suspect improved cognitive performance observed in previous exercise trials has been the result of mechanisms separate and distinct from improved circadian regulation. We expect regularly scheduled daily exercise—or at least every other day—would be necessary to have chronobiotic effects. That being said, it may be possible to enhance efficacy of exercise interventions by increasing frequency to add chronobiotic effects and improved circadian regulation—which may result in synergies that further improve sleep quality and cognitive function in MCI. Furthermore, studies are needed to confirm exercise effects vary in a time dependent manner, in the same way that light does.

##### A combined approach to chronotherapy is best practice

If improved circadian regulation in older adults at high AD risk is the objective, we wish to emphasize the importance of understanding how our biological clock processes time cues. The SCN has evolved intricate pathways capable of integrating diverse time cues (i.e., light, activity, mealtime, and social), enhancing precision of entrainment to the environment in which we live. Given age-related weakened circadian regulation—which is exaggerated in AD and perhaps in MCI—it makes intuitive sense that combining BLT, melatonin, exercise, food restriction, and improved sleep hygiene would have greatest effect. We hypothesize that for individuals at high AD-risk, regulation of circadian rhythms and sleep will be best improved by a combined, coordinated chronotherapeutic approach that employs daily BLT and exercise—in the morning and again in the evening—with low-dose melatonin supplements at night before bed, and regularly scheduled mealtimes (i.e., breakfast, lunch, and dinner while avoiding large meals at night that suppress melatonin).

**Figure 2:**
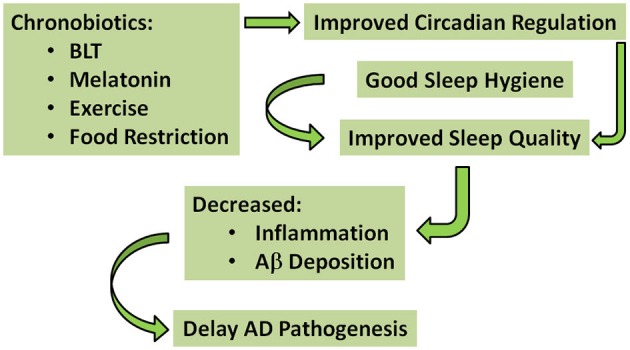
**A simplified schematic of the proposed interventions that may have potential to delay AD pathogenesis**. The green arrows indicate pathways for improved circadian regulation and sleep quality, ultimately delaying AD pathogenesis. According to this model, chronobiotics (i.e., bright light therapy (BLT); melatonin; exercise; and food restriction) and good sleep hygiene could be used individually—but preferably in combination—to improve circadian regulation and sleep quality, decrease inflammation and Aβ deposition, and thereby delay AD pathogenesis.

While we fully understand the need to test intervention components independently, we believe equal importance should be placed on assessing the potential for synergistic effects when chronobiotics are used in a coordinated fashion. Similarly, using the neuroinflammatory perspective of AD as an example, we believe it makes sense to employ multifactorial approaches—combining known anti-inflammatory factors—when developing and testing potential interventions (see Figure 2 for a schematic outlining the proposed interventions with potential to combat AD pathogenesis). As such lifestyle interventions that target improved diet, increased exercise, improved sleep hygiene, and chronotherapy would seem to provide our best option to identify an effective intervention—compared with a singular focus aimed at determining independent efficacy of any of these factors individually. Given the frightening economic impact and human costs associated with AD, we argue that our highest priority should first be to identify an approach that works—combining all of the tools at our disposal—and only after we have done so, should we turn our focus to dissecting the independent mechanisms underlying that success.

## Conflict of interest statement

The authors declare that the research was conducted in the absence of any commercial or financial relationships that could be construed as a potential conflict of interest.
